# The Role of *in silico* Research in Developing Nanoparticle-Based Therapeutics

**DOI:** 10.3389/fdgth.2022.838590

**Published:** 2022-03-16

**Authors:** Migara Kavishka Jayasinghe, Chang Yu Lee, Trinh T. T. Tran, Rachel Tan, Sarah Min Chew, Brendon Zhi Jie Yeo, Wen Xiu Loh, Marco Pirisinu, Minh T. N. Le

**Affiliations:** ^1^Department of Pharmacology and Institute for Digital Medicine, Yong Loo Lin School of Medicine, National University of Singapore, Singapore, Singapore; ^2^Immunology Program, Cancer Program and Nanomedicine Translational Program, Department of Surgery, Yong Loo Lin School of Medicine, National University of Singapore, Singapore, Singapore; ^3^Life Sciences Undergraduate Program, Faculty of Science, National University of Singapore, Singapore, Singapore; ^4^Vingroup Science and Technology Scholarship Program, Vin University, Hanoi, Vietnam; ^5^Jotbody (HK) Pte Limited, Hong Kong, Hong Kong SAR, China

**Keywords:** nanomedicine, artificial intelligence, simulation model, nanoparticle, therapy

## Abstract

Nanoparticles (NPs) hold great potential as therapeutics, particularly in the realm of drug delivery. They are effective at functional cargo delivery and offer a great degree of amenability that can be used to offset toxic side effects or to target drugs to specific regions in the body. However, there are many challenges associated with the development of NP-based drug formulations that hamper their successful clinical translation. Arguably, the most significant barrier in the way of efficacious NP-based drug delivery systems is the tedious and time-consuming nature of NP formulation—a process that needs to account for downstream effects, such as the onset of potential toxicity or immunogenicity, *in vivo* biodistribution and overall pharmacokinetic profiles, all while maintaining desirable therapeutic outcomes. Computational and AI-based approaches have shown promise in alleviating some of these restrictions. *Via* predictive modeling and deep learning, *in silico* approaches have shown the ability to accurately model NP-membrane interactions and cellular uptake based on minimal data, such as the physicochemical characteristics of a given NP. More importantly, machine learning allows computational models to predict how specific changes could be made to the physicochemical characteristics of a NP to improve functional aspects, such as drug retention or endocytosis. On a larger scale, they are also able to predict the *in vivo* pharmacokinetics of NP-encapsulated drugs, predicting aspects such as circulatory half-life, toxicity, and biodistribution. However, the convergence of nanomedicine and computational approaches is still in its infancy and limited in its applicability. The interactions between NPs, the encapsulated drug and the body form an intricate network of interactions that cannot be modeled with absolute certainty. Despite this, rapid advancements in the area promise to deliver increasingly powerful tools capable of accelerating the development of advanced nanoscale therapeutics. Here, we describe computational approaches that have been utilized in the field of nanomedicine, focusing on approaches for NP design and engineering.

## Introduction

Nanoparticle (NP)-based therapeutics have gained increased popularity in recent years. This is attributed to the many advantages that nano-formulated therapeutics such as nanoscale drug delivery systems can offer over free drugs, including the ability to bypass biological barriers with ease, improved amenability without having to chemically alter the drug and the option to direct NPs to desired target sites (1). They have been represented in many forms, ranging from synthetic polymer-based or liposomal formulations to metal or silica-based NPs and, more recently, the naturally-derived class of lipid bilayer-enclosed vesicles commonly termed extracellular vesicles (EVs). The design and engineering of these NPs has been at the forefront of many studies in the years since the introduction of these nanoscale vectors, with groups working to improve various aspects such as delivery efficacy, specificity and safety.

However, despite the surge in preclinical studies in the field, there are few NP-based drugs approved for clinical use due to unforeseen difficulties faced in numerous stages of research and development (2, 3). Data obtained from clinicaltrials.gov records thousands of trials using different classes of NP for a multitude of applications including imaging, diagnosis and drug delivery. Given the vast heterogeneity between different classes of NPs, different types of NP classes are preferably used for specific indications. For instance, lipid NPs or protein-bound NP-based therapies are frequently applied in some solid cancers, such as breast cancer, ovarian cancer, pancreatic, and lung cancer, whereas metallic NPs are more commonly used to treat infections (4, 5). More recently, natural extracellular vesicles are being explored as natural nanoscale vectors for drug delivery in the treatment of a wide range of diseases.

Instances of FDA-approved NP-based formulations for use in the clinic include ONPATTRO, VYXEOS, and NBTXR3 to treat transthyretin amyloidosis, acute myeloid leukemia and locally advanced squamous cell carcinoma, respectively (2). The success of these approaches in effect, contributed to rapid advances in NP formulation and the development of many subclasses of NPs in the clinical landscape, some of which have reached clinical trials (Table 1). Despite this, the percentage of NP-formulated drugs that successfully make it to clinical use is still staggeringly low, as highlighted in a review by Anselmo and Mitragotri (2, 6), wherein they contrasted the large numbers of trials vs. the sparse number of approved NP-based drugs. More importantly, they highlighted the challenges associated with controlling NP pharmacokinetics and pharmacodynamics *in vivo*, which traced back to the outcomes in the clinical stage, an issue also discussed by Mitchell et al. (3). As is clear from these studies, there is a gap in translating the pre-clinical efficacy of NP-based drugs to the clinical stage, an issue that underpins the need to develop more robust and translatable NP designs that can facilitate a higher rate of successful clinical translation.

**Table 1:** Various NP types used for clinical studies.

**Class**	**Diameter (nm)**	**Clinical development**				
		**Description**	**Phase**	**Target condition or disease**	**Status**	**NCT identifier**
Lipid NP	100–200	PEGylated liposomal doxorubicin	IV	Anticancer (breast neoplasms)	Completed	NCT00128778
		mRNA-lipid NP	III	Antiviral vaccine (SARS-CoV-2)	Recruiting	NCT04368728
					Active	NCT04470427
Protein-bound NP	100–300	Albumin-bound paclitaxel combined with cisplatin	IV	Anticancer (squamous cell carcinoma of head and neck)	Recruiting	NCT04766827
		Albumin-bound paclitaxel followed by anthracycline regimens	III	Anticancer (breast cancer)	Active	NCT01822314
EVs	30–1,000	Tympanoplasty with platelet- and EV-rich plasma	II/III	Anti-infective (chronic otitis media), Restoration (tympanic membrane perforation)	Recruiting	NCT04761562
		Tumor antigen-loaded dendritic cell-derived exosomes	II	Anticancer (non-small cell lung cancer)	Completed	NCT01159288
		Mesenchymal stem cell-derived exosomes	I/II	Antiviral vaccine (SARS-CoV-2), disorder (acute respiratory distress syndrome), and inflammation (SARS-CoV-2 pneumonia)	Not yet recruiting	NCT04798716
		Bone marrow mesenchymal stem cell-derived extracellular vesicles	I/II	Restoration (acute respiratory distress syndrome)	Not yet recruiting	NCT05127122
		Mesenchymal stem cell-derived exosomes and microvesicles	II/III	Restoration (Diabetes mellitus type I)	Unknown	NCT02138331
						
Polymeric NP	10–1,000	Chitosan NP with norovirus virus-like particle and monophosphoryl lipid A	I	Antiviral vaccine (Norovirus)	Completed	NCT00806962
		Dendrimer-conjugated Bcl-2/Bcl-xL inhibitor	I	Anticancer (Advanced solid tumors, lymphoma, multiple myeloma, and hematological malignancies)	Completed	NCT04214093
		Polyamidoamine dendrimer NP with pulpine	N.A. (clinical)	Restoration (Deep caries)	Completed	NCT04262076
		Holmium-166 polylactic microspheres	II	Anticancer (liver neoplasms)	Completed	NCT01612325
Metallic NP	1–100	Spherical Nucleic Acid (SNA)-based gold NP	Early I	Anticancer (gliosarcoma and recurrent glioblastoma)	Completed	NCT03020017
		Silica-gold (iron-bearing) NP and plasmonic photothermal therapy	N.A. (clinical)	Restoration (stable angina, heart failure, atherosclerosis, and multivessel coronary artery disease)	Completed	NCT01270139
		Gold NP conjugated to CD24	N.A. (clinical)	Diagnostics (carcinoma ex pleomorphic adenoma, pleomorphic adenoma)	Completed	NCT04907422
		Solution with silver NP, chitosan and fluoride	III	Anti-microbial (dental caries)	Completed	NCT03186261
		Superparamagnetic iron oxide NP	IV	Imaging (pancreatic cancer)	Completed	NCT00920023
		Novel magnetic NP with and indocyanine green	I/II	Tracking (colorectal cancer)	Not yet recruiting	NCT05092750
		Magnetic iron NP and thermoablation	Early I	Anticancer (prostate cancer)	Completed	NCT02033447
		Hafnium oxide NP (radioenhancer) with radiation therapy	II/III	Anticancer (soft tissue sarcoma of extremity and trunk wall)	Completed	NCT02379845
Silica NP	2–1,000	Fluorescent cRGDY-PEG-Cy5.5-C dots	I/II	Imaging (head and neck melanoma)	Recruiting	NCT02106598
		Photothermal ablation *via* silica NP with gold shell	N.A. (clinical)	Anticancer (prostate neoplasms)	Recruiting	NCT04240639
Quantum dots	2–10	Veldoreotide-coated CdS/ZnS core-shell type quantum dots	I	Anticancer and imaging (breast cancer, skin cancer)	Recruiting	NCT04138342
		Graphene quantum dots combined with nanowire photoelectrical immunosensor	N.A. (clinical)	Diagnostics (acute myocardial infarction)	Not yet recruiting	NCT04390490
Carbon nanotubes	0.4–40	Buckypaper	I/II	Restoration (hernia of abdominal wall, incisional hernia)	Unknown	NCT02328352

Predicting the *in vivo* behavior of NPs is a daunting task. Indeed, many factors inherent to the NP, functionalization approaches and the *in vivo* model in use could potentially affect the therapeutic outcome and safety profile. While *in vitro* delivery of NP behavior can be optimized relatively easily, these results are rarely translatable *in vivo* (7). Given the large number of variables such as route of administration, drug bioavailability, spatial and temporal targeting and the need to overcome physiological barriers, *in vivo* drug delivery remains a major challenge (8). Moreover, minute changes in the physicochemical characteristics of these complex structures can drastically alter a NP's pharmacological profile. The plethora of associated variables also make it impractical to use high throughput methods to determine optimal *in vivo* treatment conditions in most cases.

**Figure 1:**
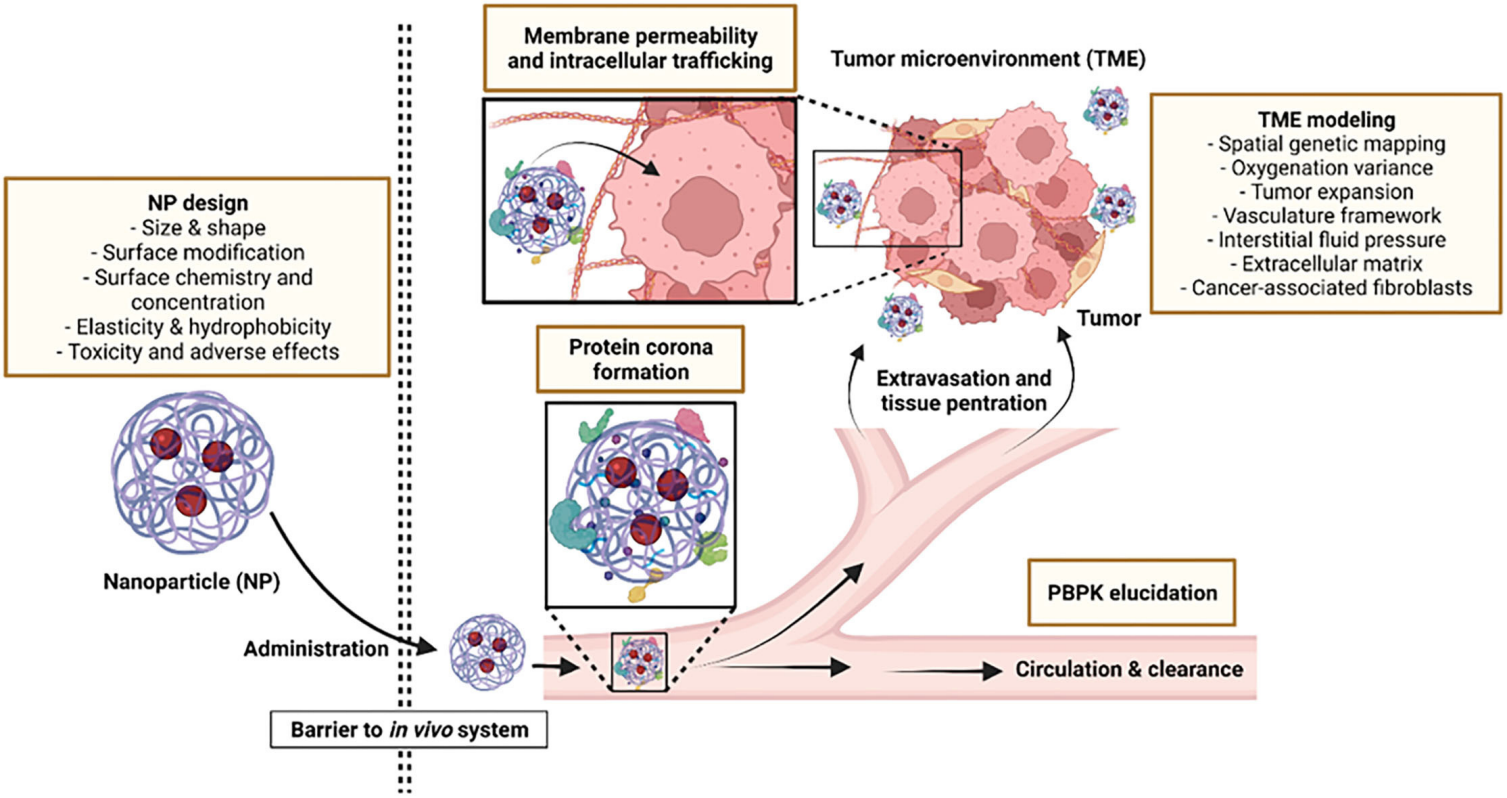
Aspects where computational modeling can be applied to improve NP design and functionality.

Exploiting technological advancements such as *in silico* modeling can help direct the design of better nanomedicine platforms while the application of novel computational approaches can expedite the process of NP development. By combining data from advanced imaging systems with computational approaches, scientists are able to interpret data more accurately, allowing them to model improved NPs and understand the impact of specific modifications on the fate of NPs upon administration. Moreover, molecular dynamic simulations can be used to develop, predict and optimize uptake of NPs with distinct physicochemical properties in *in silico* models, streamlining the research and development process to successfully guide drug and vehicle selection for clinical trials (9). Through computational learning, it is also possible to predict physiochemical properties of targets, biodistribution and quantitative assessment of NPs on the spatial scale (10, 11). In this way, many aspects of NP-based drug delivery platforms such as physicochemical-functional relationships, dose quantification for determining therapeutic and side effects and evaluation of drug efficacy over time can be predicted. Figure 1 outlines aspects of the NP design process where computational approaches have been successfully applied.

More recently, Al has allowed us to study the aforementioned challenges using *in silico* models entirely, even in areas where there is sparse data. This is possible *via* novel machine learning approaches such as deep learning, which allows computers to incorporate large data sets of established knowledge into powerful algorithms that are able to learn to efficiently and accurately predict outcomes for previously unexplored events (12–14). Together with other computational approaches, AI enables researchers to obtain a more holistic view of NPs, taking into account the multitude of interactions that mediate the properties of the NP itself (such as polydispersity or drug encapsulation efficiency) and its *in vivo* pharmacokinetic parameters, such as cellular uptake, adsorption and biocompatibility (7, 12–14). However, while AI and deep learning are extremely powerful tools, the application of true AI-based approaches in the field of NP design is still in its infancy and studies of this nature are few and far between.

Thus, in this review we focus primarily on *in silico* modeling and computational approaches that have been used in the area of NP design. Here, we refer to NPs as any structure (synthetic or natural) with at least one nanoscale dimension. The first section of this review discusses how *in silico* modeling approaches can accelerate the design of nanotherapeutics to optimize cellular interactions and uptake. The biochemical and physicochemical properties of the NP and the effect of specific NP modifications are discussed in detail in this section. We will also discuss in section three how computational modeling can be used to predict the NP's *in vivo* behavior, focusing on the aspects of tissue penetration, circulatory flux, and the tumor microenvironment (TME). In particular, we explore existing computational models and software that have been utilized for predicting specific aspects of *in vivo* NP administration and provide examples of their usage.

The promise and potential of nanotechnology, enhanced by the implementation of computational approaches, is the future of precision nanomedicine. *In silico* approaches such as those discussed in this manuscript will be instrumental in realizing the next generation of NP-based therapeutics. Hence, this review aims to put in place fundamental concepts that could further scientific investigations in this emerging field.

## Computational Approaches for NP Design

This section will discuss the computational approaches that have been implemented to study the various physicochemical properties of NPs in relation to their interactions with cell membranes, and how they may be optimized to attain efficient functional delivery of encapsulated drugs to target cells. While the utilization of computational approaches for drug design is not a new concept, *in silico* approaches for NP design differ significantly from approaches used for traditional drug design. Traditional computer aided drug discovery relies on approaches such as structure-based drug discovery of ligand-based drug discovery together with *in silico* modeling software to design drugs capable of targeting specific disease targets to exert desired therapeutic effects (15). However, computational approaches dedicated to NP-based drug design focus primarily on the interactions of NPs with cellular membranes and the how NPs could be modified to achieve desirable uptake kinetics.

Three different simulation methods are predominantly used in computational modeling of the NP's interactions with cell membranes. These include all-atom molecular dynamics (AAMD), coarse-grained molecular dynamics (CGMD) and dissipative particle dynamics (DPD). AAMD is a useful technique to obtain detailed and reliable information on simple structures at the atomic level. However, due to its low efficiency and demanding resource requirement, other methods have been used for more complex structures. This is due to the inability to simulate physiological phenomena in real-time, its limited spatiotemporal scale and its high calculation costs. Therefore, there has been a shift to the use of CGMD (16). CGMD is a simplified version of AAMD and uses the concept of beads, where each bead is a cluster of various pseudo-atoms with similar properties. Every bead can be a group of 3–5 heavy atoms. Hence, this allows the bead interactions *via* chemical properties to be modeled based on the similarities within each cluster. Although this technique is simpler, more caution is needed to reproduce factors such as the distribution of the chemical functional groups. It also only provides rough information at the atomic level. Lastly, DPD is the most simplified computational simulator amongst the three, comprising only two classifications of beads: hydrophilic beads and hydrophobic beads. With retainment of Brownian motion characteristics, it consists of overlapped beads in space. The beads' interactions are also artificially defined in this method (17).

These computational models are useful tools that enable us to better understand the interactions of NPs. However, most currently used modeling approaches are unable to take into account a holistic view of NP physicochemical characteristics, particularly with highly complicated structures such as biologically derived EVs that consist of a complex milieu of proteins, lipids, and glycans with diverse patterns of organization. Nevertheless, these modeling approaches are able to predict with high degrees of accuracy how specified parameters such as size, shape, surface chemistry (charge/hydrophobicity), or specific surface modifications can impact characteristics of simpler models of NPs. However, care should be taken when implementing these approaches as there is a great deal of interplay between these characteristics and interactions with cellular membranes and uptake is rarely dependent on a single characteristic alone.

Moreover, while computational models allow a faster rate of screening or analysis of NP properties *in silico*, care should be taken when implementing conclusions drawn from modeling approaches across different studies. Existing literature has documented a myriad of NPs with different sizes, formulations and physicochemical properties, some of which were outlined briefly in Table 1. The distinct profile of a given NP is a key factor to take into consideration when it comes to modeling NP bahavior. Empirical means have deduced that the delivery efficiency fluctuates with the material make-up of NPs with the caveat that the repertoire of synthetic, organic and natural NPs currently in use all require distinct parametric input for computation, given the large variations in their physicochemical properties. The change in the physicochemical properties of the NP would affect their behavior and function in biological systems. Therefore, prior investigation into factors that govern and differentiate NPs is a prerequisite and it should be noted that trends in NP behavior obtained with a particular NP may not be universally be applicable to all NPs.

### Size of NPs

With sizes ranging from 1 to 1,000 nm, determining the optimal size of NPs for a specific purpose is essential because it directly affects a number of important factors such as tissue permeability, interaction with cell membranes and uptake kinetics that ultimately determine the pharmacokinetics of the encapsulated drug (18). However, the optimal size of NPs is dependent on a number of different variables, including the recipient cell type, the route of uptake and the nature of the NP itself. Despite this, a number of simplified models have been developed that use computational modeling to determine how the size of various NPs can impact rates of uptake.

For instance, given that cellular uptake of NPs is highly dependent on their size (19, 20), using DPD simulation, Gao et al. (21) investigated the endocytosis dynamics upon NPs' interaction with the cellular membrane and, in particular, the impact of the NP size on this process. The authors demonstrated that, in the case of weak interactions between NPs and the membrane, a membrane bending determined the wrapping of large NPs (35.7 nm). However, oversized NPs could cause membrane disruption during penetration, which encourages scientists to optimize the NP surface by balancing translocation efficiency and toxicity to the cells (22–24).

The effect of size on NPs was also elucidated in another study by Huang et al. The authors leveraged the CGMD model to demonstrate that the size of NPs is crucial in the determination of complete endocytosis (20–22). It is thought that the wrapping process of NPs by cell membranes determines whether the endocytosis of the NPs is successful. Generally, this process is greatly dependent on size and thus, NPs have to be of a certain size for successful induction of wrapping and, subsequently, endocytosis (18). In the study conducted by Huang et al., they found that NPs of smaller sizes (*R* = *5.0* σ, where *R* is radius; σ is ~2 nm) were not effectively endocytosed as compared to NPs of a slightly larger size at *R* = *7.5* σ. However, it was also noted that NPs should not be too large.

On the contrary, another study investigated the sizes of gold NPs (AuNPs) coated with dodecanethiol, termed dodecanethiol-coated neutral hydrophobic AuNPs. The authors found that the AuNPs that had smaller sizes (~2 nm) were able to penetrate the cell membrane most efficiently, as compared to sizes of 3, 4, and 5 nm (25). However, this finding might have been attributed to the dodecanethiol, a type of surface modification that has a hydrophobic nature, thereby facilitating the entry of smaller NPs.

Another study used the DPD simulation to investigate the sizes of NPs in endocytosis. They found out that clustering multiple NPs allowed for the emergence of cooperative endocytic behavior (26). By clustering multiple NPs, the large energy barrier of overcoming the wrapping process of a single NP can be lowered. The study showed that clustering of NPs with a diameter of 2.5 nm formed close-packed aggregates and those with intermediate sizes of 4 nm were seen in a linear pearl-chain-like formation. On the other hand, larger NPs of size 6 nm do not partake in cooperative endocytosis as they were large enough to be endocytosed independently. Similar to another study, smaller NPs with diameters of 3 nm formed isotropic patch-like clusters, whereas larger NPs with diameters of 7.5 nm formed pearl chain-like structures (27). Therefore, the way that these NPs aggregate based on their size could also affect endocytosis.

### Surface Modification of NPs

Computational approaches have been used to improve the loading and retention of drugs within NPs by modifying their surface chemistry. Previous studies have shown that NPs could be primed by modifications to their surface. For example, drug loading and release can be controlled *via* silane modification of surface properties as demonstrated by Manzano et al. (28), where amine-functionalized MCM-41 micro-spheres demonstrated longer drug retention in comparison to particles that are irregularly shaped. Another common type of chemical modification to improve drug delivery includes the process known as PEGylation, a process involving the addition of a hydrophilic polymer consisting of repeated ethylene ether units of PEG that are passivated onto the NPs' surface. Jung et al. showed that this could extend the drug circulation time by preventing aggregation and protecting against phagocytosis and opsonization. Cavitation has also been utilized to obtain smaller and more stable metallic NPs that can be used for drug delivery (29, 30). In this regard, Lachaine et al. (31) created a computational framework to efficiently screen a large database of metallic structures and materials *in silico*. With this framework, the nano-cavitation ability of NPs could be identified by the prediction of the laser fluence needed to prompt cavitation around NPs made of varying materials and compositions. The article concludes that nanoshell structures consisting of silica-metals are able to decrease the cavitation threshold due to their extensive spectral tunability, thus enhancing cavitation. Sviridov et al. (32) also showed that porous silicon NPs with reduced cavitation threshold could aid in cancer cell destruction.

### Shapes of NPs

In addition to size, the NP shape also plays an important role in the interaction of NPs with phospholipid membranes, which determines the internalization efficacy (33, 34).

NPs come in all kinds of shapes or geometries, which include spheres, rods, cones, ellipsoids, discoids, and clubbed shapes. The diversity in distinct morphologies of NPs indicates that it is highly likely that the shapes of NPs are an important factor to consider for delivery. In fact, NP morphologies play a crucial role in their uptake, delivery, and distribution (35). Generally, NPs that are cylindrical tend to have increased numbers of binding sites, and therefore, the interaction between cylindrical NPs and the cell membrane often results in better uptake as compared to spherical NPs, despite having the same size (36, 37).

Utilizing the DPD simulation model, Yang and Ma (38) showed that the shape and orientation of NPs have a multiplex impact on their translocation, so that modifying the parameters can improve the interaction between NPs and cell membranes. Using a large-scale CGMD model, the effect of NP shape on the endocytosis process was shown to be rather complex (39). For instance, spherical particles were demonstrated to be easier to internalize than ellipsoidal ones due to the lower bending energy required (40). But in another example using the CG model, spherical particles appeared to facilitate less efficient delivery than same-sized spherocylinders (41). As shown previously, NPs which possessed rod-like morphologies had a high internalization rate as compared to spherical NPs. In the study, Vacha et al. (41) performed CGMD simulations using NPs in the shape of spherocylinders. A spherocylinder is a shape which is cylindrical along its length with hemispherical caps on both ends. The spherocylindrical NP was shown to be capable of assuming various spatial positions, such as being parallel or perpendicular to the plane of the cell membrane. The CGMD simulation illustrated that the spherocylindrical NP can rotate to become parallel to the cell membrane surface. After the rotation, it can further get encapsulated and subsequently endocytosed. When compared to NPs that are spherical and of the same diameter, the authors found out that spherocylinders were more favorably encapsulated.

The time of encapsulation is another factor to be considered. NPs that have elongated morphologies such as clubbed shapes tend to have higher wrapping times, thereby impeding internalization rates. Therefore, elongated NPs can have a lower uptake rate as compared to spherical NPs (42). A study has shown that NPs with a discoid morphology showed higher internalization rates into HeLa cells when compared to rod-shaped NPs, despite having similar sizes (43). In addition, a different study has elucidated that cylindrical NPs displayed a lower internalization rate as compared to spherical NPs, where these NPs were prepared *via* self-assembly of poly(acrylic) acid and polystyrene diblocks (37). Therefore, the aforementioned findings showed that spherical NPs could also display comparable internalization rates (35).

With increasing advancements in nanotechnology, newer computational platforms such as the COMSOL Multiphysics simulation software have been used in the quest to determine the best NP shapes. Through simulation, researchers found that NPs that have twinned morphology were able to deliver the highest concentrations of drugs as compared to oval-shaped NPs (44).

### Surface Chemistry of NPs

Cell membranes are negatively charged in nature, and therefore the surface chemistries of NPs are an important factor in mediating the interactions between NPs and cells. Nangia and Sureshkumar (45) used computational modeling of negatively-charged cell membranes to investigate how negative or positive charges on NPs affected NP uptake. Using a CGMD model, the authors simulated the interaction between NPs and negatively charged membranes (3:1 ratio of neutral distearoyl phosphatidylcolinel and negatively charged distearoyl phosphatidylglycerol). Accordingly, they demonstrated that positively charged NPs showed high affinities to the lipid membranes. This was further attributed to the shapes of the NPs. For instance, positively charged rice-shaped NPs were seen to cause severe disruption once they came into proximity with the lipid bilayer because they were able to orientate themselves in parallel with the lipid bilayer, which maximized adhesion and, subsequently, causing substantial membrane damage. Hence, they conclude that the interplay between shape and surface charge could affect translocation rates by 60 orders of magnitude.

Another study combined both the use of experimental methods and DPD simulation to investigate the effects of the internalization of NPs (46). Experimental results showed that NPs that were positively charged entered the cells cooperatively, although repulsion was observed between the NPs. Although the positively charged NPs were also expected to repel each other, these NPs were seen aggregating on the surface of the negatively charged cell membrane, before being endocytosed by the cell *via* a single vesicle. Subsequently, DPD simulations were conducted to confirm experimental findings. In their simulation, the authors coated small-sized NPs with positively charged ligands (diameter of 3.23 nm) and simulated using two NPs for simplicity. They found out that a ligand density of 0.1 is the minimum number to induce endocytosis (46). In addition, endocytosis is positively correlated with the density of charged ligands. The higher the density, the more likely endocytosis of the NPs will occur. However, simulations using one positively charged NP of the same size did not induce endocytosis, which proves that the internalization of these NPs is indeed a cooperative process.

### Concentration of NPs

NP concentration is a key factor affecting cellular uptake—failure to meet an optimal concentration would diminish efficient NP-mediated drug delivery. In a study conducted by Chen et al., they used DPD simulations to investigate the effects of NP concentrations on the uptake of these particles by the cells. Cooperative chain-like penetration of NPs was seen as they tend to aggregate before penetration—a finding that was also observed in Yue et al.'s study (26, 27). However, if the concentrations of the NPs are too low, the cooperative process is unable to take place, thereby leading to poorer uptake rates. On the other hand, higher concentrations of NPs will run the risk of vesicle ruptures. Hence, efficient uptake of NPs requires administration of NPs at optimal concentrations appropriate for their size. This is because smaller NPs will aggregate together to reduce the energy needed to bend the cell membrane for penetration. Therefore, at high concentrations of small NPs (diameter: <2.5 nm), they will undergo cooperative chain-like penetration where they either display the isotropic patch-like clusters or the linear pearl-chain-like clusters. On the other hand, NPs possessing diameters larger than 2.5 nm can penetrate the membranes directly, hence, NPs of larger sizes do not have to be used in high concentrations to achieve membrane penetration. Therefore, both NP concentration and size work in synergy to achieve translocation, albeit *via* different pathways.

### Elasticity of NPs

Shen et al. (47) implemented the CGMD model to demonstrate that soft NPs were able to be wrapped by the membrane faster than stiff NPs, indicating that elasticity affects their cellular uptake. This is due to the lower energy barrier that soft NPs must overcome in order to induce membrane wrapping. However, this observation was only seen in the earlier stages of membrane wrapping. The energy barrier for soft NPs increased over time due to the change in elastic energy, and thus, the membrane wrapping process gradually slowed down. Therefore, the stiff NPs eventually clocked a shorter wrapping time and were more efficiently internalized as compared to the soft NPs. Shen et al. went to further investigate the reasons why soft NPs had a slower membrane wrapping process, by similarly using the CGMD model. They found that soft spherical NPs had to recruit several more receptors *via* lipid-mediated endocytosis to drive membrane wrapping as compared to stiff NPs to overcome the deformation due to their elasticity, thereby delaying the endocytosis of the soft spherical NPs (48). The energy barrier that needed to be overcome increases as the size (radius) of the NP increases, thus making likely an interplay of the elasticity and size properties. Although this similar trend can be seen in both the soft and stiff NPs, the wrapping time for soft NPs is still higher as compared to stiff NPs, given at any size. Therefore, both elasticity and size are interlinked and would impact rates of endocytosis.

### Hydrophobicity of NPs

Cell membranes are made up of phospholipids which are arranged in a bilayer that consists of both hydrophobic and hydrophilic molecules. Thus, the amphipathic property of cell membranes challenges NP design in obtaining the optimal balance between hydrophobicity and hydrophilicity. In a study using computational DPD model, Ding and Ma (49) figured that the hydrophilic gradient of the NPs determined the translocation time of the NPs, since hydrophobic particles were more likely to insert into cell membranes. A study using CGMD simulations found that the hydrophobicity of NPs affected the interaction with the cell membrane. NPs which were fully hydrophobic were able to penetrate through the lipid portion of the cell membrane. On the other hand, NPs that were semi-hydrophilic were unable to penetrate through the cell membrane but were able to be adsorbed on the surface of the membrane (50).

One other study conducted by Gupta and Rai (51) also used CGMD to simulate skin lipid membranes with different constructs of NPs by tweaking the levels of hydrophobicity and hydrophilicity. By adjusting the ratios of both the hydrophobic and hydrophilic beads during the simulation, they found out that the best ratio was a 2:1 ratio of hydrophobic to hydrophilic beads. This ratio resulted in cell membrane penetration, followed by endocytosis through the skin cell membrane. Subsequent experiments also showed that proteins could be successfully delivered. However, other ratios used resulted in NPs being adsorbed on the surface. Therefore, a good balance of both hydrophobic and hydrophilic regions of NPs can help to facilitate the delivery of these NPs, which can be packaged with therapeutic drugs.

### Safety of NPs

Apart from improved drug efficacy, computational approaches can also be used to improve the safety of the NPs. A article by Burello showcased a computational method to design safe yet functional NPs (52). This method sifts out functional core structures that meet the functionality requirements yet have the lowest toxicity from a library of potential NPs. These core structures were then coated with a biocompatible metal oxide shell, called core@shell in the article, which protected the reactive core material from ultraviolet light. The core@shell particles were scored based on a list of physicochemical properties to identify potential shell materials. Based on the scoring, silica was identified as a promising material for drug delivery systems and biosensors. This system was proven useful in anticipating the safety of the materials in the R&D phase, making it more efficient to remove those that do not meet the requirements. Apart from safety, these computational core-shell systems can be used to increase the catalytic activity through a continuum-based strategy to screen the surface strains (53). By accurately screening the surface strains and creating core-shell models with the least input, they showed the system's ability to control the shell thickness, as well as the NPs' core sizes.

## Computational Approaches to Predict *in vivo* Behavior of NPs

As we have described before, NPs present many advantages when used as drug carriers, as they are able to reduce the side effects of many drugs, increase drug half-life in circulation and improve drug accumulation at the target sites inside the body (54, 55). However, there are conflicting reports on the *in vivo* therapeutic efficacy of NP-based drugs, with a previous study indicating that NP-based delivery resulted in relatively low delivery efficiency (56). This is corroborated by the fact that there are only a few NP-based therapeutics available for clinical use (2). The key reason for these failures is most likely the unpredictable barriers in the *in vivo* environment and the heterogeneity amongst patients leading to inefficient delivery of certain drug loads to the target sites. To overcome these challenges, it is necessary to fully understand the scope of NP behavior *in vivo* and their interactions with a multi-dimensional environment, rather than solely considering isolated factors; as much as *in vitro* experimentation and empirical derivations can be measured in a quick and manageable manner, correlation of these models with their more complex *in vivo* counterparts is difficult to achieve. Moreover, translation of *in vitro* findings and measurements onto *in vivo* bodies would often be an erroneous extrapolation, proving translational treatments to be difficult. Fundamentally, *in vitro* and *in vivo* environments are distinct; a controlled *in vitro* environment can be calibrated to allow NP and therapeutics alike to function optimally, while their introduction into *in vivo* bodies would in essence strip most external control out of our hands.

Generally, NPs face problems when applied in biological systems, such as inevitable interaction with the immediate surrounding biomolecules when administered to the vasculature. Given the stark difference between *in vitro* and *in vivo*, compounded with the technical barrier to readily measure NP activity or function in biological systems, turning to computational models may elucidate and shed some light on problems stemming from NP use *in vivo*. Computational models, which can simulate the effects of NP properties (size, shape, surface functionalization, stiffness, composition, drug loading and retention, etc.) on their interaction with the *in vivo* environment, can help to reduce cost and speed up *in vivo* optimization. More than just simulating the characteristics of the particles, a multi-parameter computational model can also comprehensively and simultaneously take into consideration most factors present in an actual biological environment that influence the outcome of drug delivery. Table 2 consists of potential tools for simulation and visualization of NP behavior at different scopes of NP-environment interactions. Intrinsically, these platforms can be used to establish multi-parameterized modeling in the future, for achieving a more representative biological environment. In conjunction, interactions between NPs and various *in vivo* barriers and their subsequent effects on NP fate will be discussed in this section.

**Table 2:** Potential simulation tools for modeling NP *in vivo* behavior.

**Overarching platform**	**Software**	**Modeling allowance**	**Open-source for academic use**	**Dimension**	**Scale**	**References/Weblink**
Molecular dynamics	Desmond	All-atom molecular dynamics, coarse-grained molecular dynamics, dissipative particle dynamics	Open	2D/3D	Simulations with molecular dynamics, allowing atomic, and molecular level analyses for elucidation on physicochemical outcome	https://www.schrodinger.com/products/desmond
						https://www.deshawresearch.com/resources_desmond.html
	GROMACS		Open	2D	Highly supported and highly efficient simulation systems available	https://www.gromacs.org/ (57, 58)
Cell-based models	TumourSimulator	On-lattice	Open	3D	Cell and tissue (e.g., cell division, intratumor heterogeneity) [modeling up to 10^9^ cells]	https://www2.ph.ed.ac.uk/~bwaclaw/cancer-code/ (59)
	CompuCell 3D		Open	2D/3D	Generic cellular mechanisms (e.g., cell adhesion, division, haptotaxis and chemotaxis) [modeling up to 10^5^ cells]	https://compucell3d.org/ (60, 61)
	Chaste		Open	2D/3D	Multicellular modeling (e.g., angiogenesis, tumor growth, intra- and extravascular transportation, cell proliferation in complex 3D geometries) [able to modeling more than 10^6^ cells]	https://www.cs.ox.ac.uk/chaste/ (62, 63)
	Tumopp		Open	2D/3D	Cell and tissue (e.g., cell division, intratumor heterogeneity) [able to modeling more than 10^4^ cells]	https://github.com/heavywatal/tumopp
						https://heavywatal.github.io/tumopp/ (64)
	CellSys	Off-lattice	Closed	2D/3D	Multi-cellular systems (e.g., cell-cell, cell-matrix interaction, cell growth and migration, cellular environment) [Able to modeling more than 10^6^ cells]	http://msysbio.com/ (65)
	PhysiCell		Open	2D/3D	Multicellular systems, tissue (e.g., cell cycling, apoptosis, necrosis, solid and fluid volume changes, mechanics, and motility) [able to modeling more than 10^6^ cells]	http://physicell.org/ (66)
	Biocellion		Closed	2D/3D	Multicellular systems (e.g., cell behavior, extracellular environment, pair interaction) [able to modeling more than 10^9^ cells]	https://biocellion.com/ (67)
	IBCell		Open	2D/3D	Multicellular structure development (e.g., cell membrane, cell-cell and cell-environment interaction) [modeling a small number of cells in detail]	https://tanakas.bitbucket.io/lbibcell/index.html (68–70)
Stochastic biochemical reaction simulations	Smoldyn	Multiscale reaction-diffusion simulations	Open	3D	Cellular biochemical processes with spatial and stochastic detail (e.g., cell membrane, subcellular structures, or individual molecule diffusion, molecule-membrane interactions and chemical reactions) [modeling a small number of cells in detail]	https://www.smoldyn.org/ (71, 72)
	STEPS	Stochastic reaction-diffusion simulations	Open	3D	Cellular reaction–diffusion systems.	http://steps.sourceforge.net/STEPS/default.php (73, 74)
	URDME		Open	2D/3D	General simulations and modeling for stochastic reaction-transport.	http://urdme.github.io/urdme/ (75)
Multi-feature platforms	Morpheus	Multiscale (deterministic, stochastic reactions, spatial stochastic, hybrid deterministic/stochastic, and agent-based)	Open	2D/3D	Models interactions between discrete cells [modeling up to 10^5^ cells]	https://morpheus.gitlab.io/ (76)
	VirtualCell		Open	2D/3D	Cell membrane and subcellular structures in high spatial resolution or modeling individual molecule (e.g., molecules diffuse, interaction with surfaces, and their chemical reactions) [modeling a small number of cells in detail]	https://vcell.org/ (77, 78)

### Modeling Circulation and Clearance

Immediately following *in vivo* administration, NPs come into contact with biological fluids, resulting in the formation of a protein corona that covers the NP. This phenomenon essentially coats the surface of the NP and affects their surface functionality. As shown by Palchetti et al. (79), mimicking *in vitro* and *in vivo* incubations of PEGylated liposomes for protein corona interaction yielded a different composition of the corona. Different protein coronas in different types of NPs would in turn affect their biodistribution and circulatory flux in dissimilar manners (80). With immaculate detail, molecular dynamic simulations can capture protein corona formation and their interactions, such as protein binding competition, in the form of AAMD (81). A study by Ramezani and Rafii-Tabar (82) simulated the adsorption of human serum albumin (HSA) onto AuNPs, which in turn led them to uncover key docking amino acids (being Lys464, Thr504, Phe505, and Lys464) on HSA and highlighted the event of HSA primary denaturation. Nevertheless, the more multi-protein-NP interactions that full atomistic models can capture, the closer it is to resembling *in vivo* conditions, while the rigor required exponentially increases. The counterpart to full atomistic models (AAMD)—a coarse-grained model (CGMD)—can also capture these aspects without the luxury of rendering simpler molecular complexes; details such as atomic structure or polarity would be excluded in a coarse-grain model. A study by Hu et al. (83) using a CGMD approach had found that in the pulmonary surfactant layer, the simulated inhalation of aerosolized NPs of different charges allowed different surfactant proteins to specifically adsorb onto the NPs. Another study by Lopez and Lobaskin (84) with a CGMD approach of protein corona formation, showed possible application of their model in predicting plasma protein adsorption onto hydrophobic NP surfaces.

Software tools on molecular dynamics could be an avenue to predict molecular corona formation on NP (Table 2). Along this chain, Pal et al. (85) managed to illustrate *in silico* binding of Gemcitabine to AuNP *via* Desmond, a software capable of visualizing interactions with NP, due to its allowance on both atomic and molecular level of analyses. Ultimately, the protein corona of the NPs affects their subsequent physicochemical properties, and thus molecular exchanges with the biological system. Hence, this calls for a further traction of NP corona elucidation, which further helps to uncover more *in vivo* NP behavior.

As part of circulation, it is necessary to also consider cellular components of the blood, which have been shown to greatly impact the mobility of NPs in circulation. As a result, the design of NPs should be optimal for them to drift to the vessel wall, which facilitates the chance that NPs can penetrate the blood vessel wall and migrate to the target tissues. In circulation, red blood cells (RBCs) are separated from each other in the blood flow but cluster to form a layer in the center, leaving a cell-free layer (CFL) close to the blood vessels. For computation, modeling fluid flow with rigid bodies (RBCs, NPs, etc.) is a Fluid-Structure Interaction (FSI) problem. Employing Immersed Finite Element Method, Lee et al. studied the influence of spherical NP size on their interaction with RBCs in vascular transport, on how NPs tend to move in the CFL, a phenomenon called “margination” (86). Their work inferred that without RBCs, NPs only flowed along with the circulation without lateral drift, but had a propensity to move away from this middle stream in the presence of 15–30% hematocrit. In addition, they also found that the larger NPs (1,000 nm) were concentrated close to the vessel walls while the smaller NPs (200 nm) preferred to flow with the middle layer. Lee et al. (86) also characterized the influence of hematocrit concentration on particle dispersion. In another work utilizing combinations of DPD and smoothed DPD methods by Müller, Fedosov and Gompper, the margination of NPs was found to be highly dependent on the particle size and shape (87). Specifically, they found that micro-size particles marginated better and could deliver more efficiently than sub-micron carriers. With even more detail, the simulation model indicated that the ellipsoidal particles were less likely to bear the drag pressure from circulation flow, making them a better choice for delivery.

Interweaved with circulation, the *in vivo* pharmacokinetic profile of NPs can be formulated quantitatively, in the form of physiologically based pharmacokinetic (PBPK) modeling. However, the intrinsic differences between NP classes, such as the material make-up and physicochemical properties, would call for each NP (sub)class to have its own specific PBPK models, with tuned parameters according to their Absorption, Distribution, Metabolism, and Excretion (ADME) profiles. Hence, different PBPK models are required for different kinds of NPs (88). For instance, ADME and toxicokinetic profiles of nanoparticulate silver allowed Bachler et al. (89) to construct a PBPK model applicable for silver NPs, which allows the elucidation of silver NPs *in silico*.

To justify the computational power required for modeling circulation and clearance, it is necessary to acknowledge that this problem is compounded by the prevalence of NP variety (hence their PBPK variability). In this regard, the computationally demanding, yet beneficial step forward for an even more thorough exploration of protein corona, circulation and clearance, would be a merger of functions, as performed by software tools on molecular dynamics—(Table 2) on FSI—such as COMSOL Multiphysics (https://www.comsol.com/comsol-multiphysics) and Converge CFD (https://convergecfd.com/)—to derive a merger with specific PBPK parameterization tailored for specific NP types in the future.

In addition to the points discussed above, NP circulation and mobility is further impeded through clearance mechanisms, such as by circulating immune cells or macrophages in the liver and spleen (90–93). Nevertheless, effort has been made to steer NPs away from immune interference and increase their circulation time, through surface modification *via* CD47 coating (94), lactose coating (90), and PEGylation to mitigate NP opsonization (95, 96).

The discussed points *vide supra* are aspects that need to be integrated and considered for more accurate *in silico* modeling of NP's behavior under circulation and clearance. It is introspective that future computational models and software could be galvanized to spearhead in this direction.

### Modeling Extravasation and Tissue Penetration

Following the NPs' escape from the circulation, extravasation into tissues takes place. Extravasation of NPs out of capillaries and into tissues is central to NP-based therapeutics, as it allows the NPs to come into contact with target cells. This section discusses attempts at modeling NP extravasation and approaches used to model the interactions between NPs and *in vivo* barriers such as the endothelium.

In this respect, a study by Moradi et al. (97) demonstrated that positively charged elongated NPs were capable of crossing the endothelial barrier more easily than other particles due to electrostatic interactions. Furthermore, the NPs' exit from the vasculature to tissues was affected by particle geometry, surface chemical properties, endothelial vessel walls and local hydrodynamics conditions. To add on, Shah et al. (98) had shown that with their Brownian Dynamics simulation, particles of varying geometries (prolate spheroids, oblate spheroids, and spheres) would have drastically different extravasation rates in different flow conditions. Following the extravasation of NPs into tissues, the NPs encounter a host of additional factors that mediate uptake kinetics by tissue-resident cells. Given the vast differences in architecture and cellular composition between different tissues, it is reasonable to assume that these factors vary based on the type of tissue and the presence of other determinants such as disease or inflammation. In this subsequent portion, computational approaches for modeling NPs' delivery efficiency in anti-tumorigenic studies will be discussed within the scope of tissue penetration.

Wilhelm et al. (56) analyzed NP delivery efficiency to solid tumors by collating data from studies conducted from 2005 to 2015 and showed that the median percentage of administered NPs that reached their target tumors was ~0.7%. Thus, increasing this efficiency of delivery is a major hurdle for the translational application of NPs for the treatment of solid tumors. Currently, active or passive targeting can be used to direct NPs to tumors. Regardless of the mode of targeting, better knowledge of the tumor and its environment can facilitate improved delivery efficiency to solid tumors. Computational models allow consideration of certain key properties of tumors for modeling. Importantly, tumor cells are not isolated elements that can be looked at individually; the TME affects many aspects of a tumor and its proliferative and metastatic capabilities can also greatly impact NP behavior. Overall, the intrinsic tumor and TME properties are spatially heterogeneous or non-uniform in genetic, chemical and physical landscapes.

Another aspect to be considered with respect to NP delivery in the TME is the concept that solid tumors exert an enhanced permeability and retention (EPR) effect, which allows for passive targeting of NPs to the tumor; this EPR effect can be altered based on certain NP properties. NP size, ionic charge types and aspect ratio can influence the EPR effect, which will alter passive targeting efforts. Modeling of the EPR effect requires computation of extravasation, diffusion and convection of the NPs. However, even these factors together are insufficient to precisely predict the effect of the EPR effect, as noted by Subhan et al. (99), who reported that there are other parameters that might contribute to the EPR effect on NP extravasation, which are not yet known.

Paradoxical to the EPR effect of tumors which can be exploited for passive targeting, tumors have higher interstitial fluid pressure. After extravasation, deep penetration of the NPs to the tumors can be deflected due to the resistance faced by higher interstitial fluid pressure. LoCastro et al. (100) concluded that computational fluid modeling based on dynamic contrast-enhanced magnetic resonance imaging (DCE-MRI) could allow DCE-MRI to be a feasible interstitial fluid pressure (and velocity) modeling apparatus in neck nodal metastases. It is important to explore this area, as this affects the dose of NPs required for optimal therapeutic efficacy against the tumor.

Attributed to angiogenesis and invasive growth and/or migration of tumors, the neovasculature encapsulating tumors is spatially irregular, differing from the regular vascular network of a tumor-absent biological system. Angiogenesis leads to differential circulation of blood in different parts of the tumorigenic tissue. One consequence that cells within a tumor face as a result of this is hypoxia variance, a microenvironmental factor which can also generate tumor heterogeneity (101). Hypoxic areas develop where oxygen consumption outpaces oxygen supply. Previous attempts to explore the oxygenation status of tumors were done through biomarkers or polarographic needle electrodes, which do not provide spatial information. As noted by Powathil et al. (102), exploitation of the knowledge of the vascular environment around or in the tumor could allow *in silico* simulation on the tumor oxygenation status. Taking into consideration the oxygenation status of cancer cells is crucial as it affects hypoxia-responsive NP functionality. Hence, spatial-temporal models that include the interplay of the components of a dynamic vascular network, with simultaneous depiction of tissue models, could shed some light on the tumor oxygenation status. Welter and Rieger (103) managed to generate a discrete yet dynamic blood vessel network simulation, along with other continuous equations that accounted for heterogeneous parameters such as oxygen levels and interstitial fluid flow.

Presently, physically fabricated microfluidic systems can achieve a degree of modeling for NP extravasation, and vasculature variance for generating EPR effect, in the form of on-a-chip systems. For instance, Wang et al. (104) used a microfluidic Tumor-Vasculature-on-a-Chip, to investigate the influence of EPR effect on PEGylated and PEG-PLGA NP extravasation and selective accumulation in parts of tumors. Another similar study was performed by Vu et al. (105), which identified a correlation for greater occurrence of extravasation (and tumor penetration) with NP at 40 nm in size, or NPs functionalized with tertiary amines exhibited limited tumor penetration, as compared to those with carboxylic acid-surfaced NPs which would be able to improve tumor penetration. The data from studies like this facilitate the identification of determinants that need to be taken into consideration for developing holistic *in silico* models, that would most likely make future investigations into these microfluidic interactions more accessible. Moreover, incorporation of additional parameters such as interstitial fluid pressures, oxygenation status and mapping of vasculature could provide a more robust framework for simulating NP behavior in the TME (106).

Narrowing it down, tumors can be made up of different cell types to produce a spatially diverse genetic landscape, which can be attributed to the Cancer Stem Cell Hypothesis (107). Modeling tumor growth with a heterogeneous cellular population with cell-based models would thus be beneficial due to simulations of the spatial positions of the cancer cells, which can elucidate how the function and behavior of NPs change. Traditionally, the Eden Lattice Model was constructed to examine the two-dimensional architecture of cell growth. Today, this principle has been extended to construct various cell-based models. Broadly, depending on the discretization of defined cellular space, focus of spatial states of cells over time, and the scope (individual cells, tissues, populations, etc.), these models are either categorized to have on-lattice (lattice-based) or off-lattice (lattice-free) framework. On-lattice approach allows visualization on the overall evolution of tissue dynamics (108), such as TumourSimulator, CompuCell 3D, Chaste and Tumopp, which could provide visual representations of spatial heterogeneity (Table 2). Off-lattice approach allows the ability to describe details at the level of individual cells (109), such as CellSys, PhysiCell, Biocellion, and IBCell (Table 2). These tools allow the simulation of multicellular systems, along with the function to account for cell-cell interactions and morphology. Regardless on the divergence of foci and framework, cell-based models can be used to visualize and understand the heterogeneous landscape of tumors. Waclaw et al. (59) cell-based model simulated heterogeneous growth and elucidated the temporal evolution of the genetic landscape of the tumor, affected by factors such as cell dispersal rates and emergence and/or takeover by an advantageous mutation. Next, Tsompanas et al. (110) incorporated *in silico* optimization of NP design parameters in PhysiCell, which allowed for the team to conclude that tumors exposed with different NP types carrying the same therapeutics was most effective early in the simulation; their results could advise on period of dosing and therapies to attain optimal NP treatment by combating the dynamisms of tumors.

Hence, even with ample knowledge of the cellular landscape, barren consideration of the latter's inclusion in these models, undoubtedly over-simplifies the problem of understanding how NP behavior can be inflected. Besides the factors outlined above, other notable players affect tumor growth and subsequently NP performance. Endogenous NPs can naturally arise from cancer cells and be selectively taken up by other cancer cells; these NPs with lower internal pH can compete or interfere with administered functionalized NPs and lower their efficacy. Moreover, cancer-associated fibroblasts (CAF) modulate the physical properties of the extracellular matrix (111, 112), which alters tumor growth and metastasis. Prior work has been done to effectively disrupt their influence on tumor growth (113, 114). Hence, it is interesting to investigate endogenous-exogenous NP dynamics and CAF-silencing strategies or modeling approaches, as alternative anti-tumorigenic avenues in the future.

### Modeling Cell Membrane Permeability

The ability to cross the cellular membrane safely and efficiently is a key feature for most NP-based formulations. In spite of continuous advances in the field of nanotechnology, the inability to precisely control the NPs' entry/trafficking into cells still remains the major obstacle hampering successful clinical translation. Given the complexity of the cellular membrane, scientists have made considerable efforts to understand the biological pathways behind NP-cell interactions, NP cellular internalization and the intracellular trafficking of NPs upon cellular internalization (115). As mentioned in section Computational Approaches for NP Design, the ability of NPs to interact with and penetrate cellular membranes is mostly driven by their physical properties including shape, size, and surface charge, and therefore precise tuning of these properties can critically improve the NPs' performance (116).

It is well-known that NPs enter cells *via* either direct penetration or endocytosis (117). In this respect, the NP's size significantly impacts the route of entry, with NPs of very small dimensions preferentially adopting direct penetration (118, 119). Although this phenomenon has also been described for NPs larger than 500 nm (120), the ability to form nano-pores on the cell membrane and thereby bypass the endolysosomal system and directly enter the cytosol is a peculiarity of small NPs (10–500 nm) (121–123). In this regard, an interesting study used a mesoscale thermodynamic model to investigate the relationship between several factors including NP size, hydrophobicity and surface charge density and the formation of pores on a simulated cell membrane (22).

The term endocytosis refers to a heterogeneous group of energy-dependent mechanisms that are mainly used by macro-molecular NPs to enter the cells (124). Briefly, the NP interacts with a receptor on the cell membrane that activates a downstream cascade responsible for the uptake of the NP into the cell. The NP uptake is mediated by cell membrane characteristics, such as surface receptors (type, diffusion, and density), lipid composition, charge, membrane surface tension and membrane rigidity. However, following endocytosis, most NP-based cargo still needs to reach the cytoplasm where it can function. Thus, studying the intracellular trafficking of NPs is of pivotal importance when a given NP is meant to be used as a delivery system for therapeutic cargoes whose target is in the cytosol, such as the nucleus or mitochondria. Therefore, understanding and modeling endosomal escape strategies is pivotal for designing NPs capable of efficient cytoplasmic delivery (125–127).

Table 2 outlines multi-feature platforms and modeling tools that can be utilized to explore and optimize NP design to achieve efficient cellular permeation and functional cargo delivery through modeling of cellular permeation and intracellular trafficking of NPs. In summary, the cellular permeability and cytoplasmic cargo delivery ability of a NP is a complex biological process that is often overlooked in NP design and requires further investigation and optimization. The lack of comprehensive understanding of intracellular NP behavior and the obscure mechanisms of endosomal escape hinders significant progress in this area. However, improved understanding of intracellular trafficking and powerful modeling programs that can predict endolysosomal progression of cargo could hold the key to solving this bottleneck in successful NP design.

## Conclusion and Future Directions

Nanomedicine and AI are well-distinguished disciplines that may meet to share a common goal: to benefit human health.

Nanomedicine adopts a collection of sophisticated and smart nano-carriers that offer many advantages over traditional “free” drugs, such as improved stability, enhanced targeting activity and high tissue penetration, among others. In the present day, the use of nanomedicine is revolutionizing our approach to diagnose, prevent, and treat human diseases.

However, as discussed in this review, progressing new nanocarriers through the development pipeline is timely, costly and tedious. In addition, the *in vivo* performance of the majority of the new technologies rarely meets the initial expectation observed during *in vitro* testing. As a matter of fact, we have limited knowledge about the relationship between biology and NP-based technologies, as there are countless factors that could potentially affect the performance of nanocarriers when applied in *in vitro* or *in vivo* models. While we are able to tune the characteristics of the nanocarrier to maximize its efficiency upon interaction with a singular cell model, it is far more complicated to control its behavior in a complex living animal model, where the exact desired physical and chemical properties of the nanocarrier are key factors to ensure safe and efficient drug delivery. Given the above limitations, it is not surprising that the majority of the new nanomedicine-based technologies never progress toward the clinical setting.

Computational methods rely on experienced insights and patterns from large sets of data to support decision-based medical tasks. In this regard, progressive enhancements in computational power, as well as the corroborated reliability of numerous simulation software programs, encourage us to harness the properties of these virtual shortcuts to minimize costly screening and trial-and-error design methods and to predict the *in vivo* behavior of nanocarriers.

To date, we can access an extensive variety of computational methods that differently assist the nanocarrier development workflow. However, the lack of the parallel use of different computational tools might raise doubts regarding their reliability in assisting decision-making. Hypothetically, to generate a comprehensive prediction model of the behavior of NPs, we should adopt a large set of complementary computational aids that can differentially decode the input data from relevant research fields to provide a holistic view of NP performance. This may be possible in the future with the application of deep learning and AI-based approaches that will be able to more accurately provide an integrated view on NP design.

In addition, the ultimate goal is for the nanocarrier to be substantially efficacious to achieve clinical translation. In this regard, computational engineers, scientists, and medical doctors should actively collaborate to accelerate the development of personalized NP-based therapeutics that can overcome challenges faced by existing nanoformulations. Being able to anticipate and prevent the pitfall of NPs under specific circumstances using computational modeling would enormously empower researchers. This can streamline the NP design process, allowing researchers to optimize nanocarriers for specific applications with greater ease.

It is difficult to discern how far we are from reaching this outcome. However, both nanomedicine and computational aids have recently made tremendous progress toward this pivotal goal.

## Author Contributions

ML: conceptualization, writing, review and editing, visualization, supervision, and funding acquisition. MP: conceptualization, writing original draft, review and editing, visualization, and supervision. MJ: writing original draft, review and editing, supervision, and visualization. CL: writing original draft and review and editing. TT, RT, SC, BY, and WL: writing original draft. All authors contributed to the article and approved the submitted version.

## Conflict of Interest

ML was scientific co-founders and advisors of Carmine Therapeutics. MP was employed by Jotbody (HK) Pte Limited. The remaining authors declare that the research was conducted in the absence of any commercial or financial relationships that could be construed as a potential conflict of interest.

## Publisher's Note

All claims expressed in this article are solely those of the authors and do not necessarily represent those of their affiliated organizations, or those of the publisher, the editors and the reviewers. Any product that may be evaluated in this article, or claim that may be made by its manufacturer, is not guaranteed or endorsed by the publisher.

## References

[1] Dang XTT Kavishka JM Zhang DX Pirisinu M Le MTN. Extracellular vesicles as an efficient and versatile system for drug delivery. Cells. (2020) 9:E2191. 10.3390/cells9102191 33003285 PMC7600121

[2] Anselmo AC Mitragotri S. Nanoparticles in the clinic: an update. Bioeng Transl Med. (2019) 4:e10143. 10.1002/btm2.10143 31572799 PMC6764803

[3] Mitchell MJ Billingsley MM Haley RM Wechsler ME Peppas NA Langer R. Engineering precision nanoparticles for drug delivery. Nat Rev Drug Discov. (2021) 20:101–24. 10.1038/s41573-020-0090-8 33277608 PMC7717100

[4] Chatin B Mevel M Devalliere J Dallet L Haudebourg T Peuziat P. Liposome-based formulation for intracellular delivery of functional proteins. Mol Ther Nucleic Acids. (2015) 4:e244. 10.1038/mtna.2015.17 26102064

[5] Akbarzadeh A Rezaei-Sadabady R Davaran S Joo SW Zarghami N Hanifehpour Y. Liposome: classification, preparation, and applications. Nanoscale Res Lett. (2013) 8:102. 10.1186/1556-276X-8-102 23432972 PMC3599573

[6] Anselmo AC Mitragotri S. Nanoparticles in the clinic. Bioeng Transl Med. (2016) 1:10–29. 10.1002/btm2.10003 29313004 PMC5689513

[7] Ekins S Puhl AC Zorn KM Lane TR Russo DP Klein JJ. Exploiting machine learning for end-to-end drug discovery and development. Nat Mater. (2019) 18:435–441. 10.1038/s41563-019-0338-z 31000803 PMC6594828

[8] Haddish-Berhane N Rickus JL Haghighi K. The role of multiscale computational approaches for rational design of conventional and nanoparticle oral drug delivery systems. Int J Nanomedicine. (2007) 2:315–31. 18019831 PMC2676650

[9] Wang W Yan X Zhao L Russo DP Wang S Liu Y. Universal nanohydrophobicity predictions using virtual nanoparticle library. J Cheminform. (2019) 11:6. 10.1186/s13321-019-0329-8 30659400 PMC6689884

[10] Brown MR Hondow N Brydson R Rees P Brown AP Summers HD. Statistical prediction of nanoparticle delivery: from culture media to cell. Nanotechnology. (2015) 26:155101. 10.1088/0957-4484/26/15/155101 25797791

[11] Ng TSC Garlin MA Weissleder R Miller MA. Improving nanotherapy delivery and action through image-guided systems pharmacology. Theranostics. (2020) 10:968–97. 10.7150/thno.37215 31938046 PMC6956809

[12] Mekki-Berrada F Ren Z Huang T Wong WK Zheng F Xie J. Two-step machine learning enables optimized nanoparticle synthesis. NPJ Comput Mater. (2021) 7:55. 10.1038/s41524-021-00520-w

[13] Harrison PJ Wieslander H Sabirsh A Karlsson J Malmsjö V Hellander A. Deep-learning models for lipid nanoparticle-based drug delivery. Nanomedicine. (2021) 16:1097–110. 10.2217/nnm-2020-0461 33949890

[14] Midtvedt B Olsén E Eklund F Höök F Adiels CB Volpe G. Fast and accurate nanoparticle characterization using deep-learning-enhanced off-axis holography. ACS Nano. (2021) 15:2240–50. 10.1021/acsnano.0c06902 33399450 PMC7905872

[15] Shaker B Ahmad S Lee J Jung C Na D. *In silico* methods and tools for drug discovery. Comput Biol Med. (2021) 137:104851. 10.1016/j.compbiomed.2021.104851 34520990

[16] Charchar P Christofferson AJ Todorova N Yarovsky I. Understanding and designing the gold-bio interface: insights from simulations. Small. (2016) 12:2395–418. 10.1002/smll.201503585 27007031

[17] Liu MB Liu GR Zhou LW Chang JZ. Dissipative particle dynamics (DPD): an overview and recent developments. Arch Computa Methods Eng. (2014) 22:529–56. 10.1007/s11831-014-9124-x

[18] Meng X Li X. Size limit and energy analysis of nanoparticles during wrapping process by membrane. Nanomaterials (Basel). (2018) 8:899. 10.3390/nano8110899 30400180 PMC6266830

[19] Jin H Heller DA Sharma R Strano MS. Size-dependent cellular uptake and expulsion of single-walled carbon nanotubes: single particle tracking and a generic uptake model for nanoparticles. ACS Nano. (2009) 3:149–58. 10.1021/nn800532m 19206261

[20] Chithrani BD Chan WCW. Elucidating the mechanism of cellular uptake and removal of protein-coated gold nanoparticles of different sizes and shapes. Nano Lett. (2007) 7:1542–50. 10.1021/nl070363y 17465586

[21] Gao X Dong J Zhang X, The effect of nanoparticle size on endocytosis dynamics depends on membrane-nanoparticle interaction. Mol Simul. (2014) 41:531–7. 10.1080/08927022.2014.896995

[22] Ginzburg VV Balijepalli S. Modeling the thermodynamics of the interaction of nanoparticles with cell membranes. Nano Lett. (2007) 7:3716–22. 10.1021/nl072053l 17983249

[23] Gurtovenko AA Anwar J Vattulainen I. Defect-mediated trafficking across cell membranes: insights from *in silico* modeling. Chem Rev. (2010) 110:6077–103. 10.1021/cr1000783 20690701

[24] Ding HM Ma YQ. Computational approaches to cell-nanomaterial interactions: keeping balance between therapeutic efficiency and cytotoxicity. Nanoscale Horiz. (2018) 3:6–27. 10.1039/C7NH00138J 32254106

[25] Gupta R Rai B. Effect of size and surface charge of gold nanoparticles on their skin permeability: a molecular dynamics study. Sci Rep. (2017) 7:45292. 10.1038/srep45292 28349970 PMC5368607

[26] Yue T Zhang X. Cooperative effect in receptor-mediated endocytosis of multiple nanoparticles. ACS Nano. (2012) 6:3196–3205. 10.1021/nn205125e 22429100

[27] Chen X Tian F Zhang X Wang W. Internalization pathways of nanoparticles and their interaction with a vesicle. Soft Matter. (2013) 9:7592–600. 10.1039/c3sm50931a

[28] Manzano M Aina V Areán CO Balas F Cauda V Colilla M. Studies on MCM-41 mesoporous silica for drug delivery: effect of particle morphology and amine functionalization. Chem Eng J. (2008) 137:30–7. 10.1016/j.cej.2007.07.078

[29] Patil MN Pandit AB. Cavitation - a novel technique for making stable nano-suspensions. Ultrason Sonochem. (2007) 14:519–30. 10.1016/j.ultsonch.2006.10.007 17207650

[30] Sivakumar M Tang SY Tan KW. Cavitation technology - a greener processing technique for the generation of pharmaceutical nanoemulsions. Ultrason Sonochem. (2014) 21:2069–83. 10.1016/j.ultsonch.2014.03.025 24755340

[31] Lachaine R Boulais É Rioux D Boutopoulos C Meunier M. Computational design of durable spherical nanoparticles with optimal material, shape, and size for ultrafast plasmon-enhanced nanocavitation. ACS Photonics. (2016) 3:2158–69. 10.1021/acsphotonics.6b00652

[32] Sviridov AP Osminkina LA Nikolaev AL Kudryavtsev AA Vasiliev AN Timoshenko VY. Lowering of the cavitation threshold in aqueous suspensions of porous silicon nanoparticles for sonodynamic therapy applications. Appl Phys Lett. (2015) 107:123107. 10.1063/1.4931728

[33] Champion JA Mitragotri S. Role of target geometry in phagocytosis. Proc Natl Acad Sci U S A. (2006) 103:4930. 10.1073/pnas.0600997103 16549762 PMC1458772

[34] Chithrani BD Ghazani AA Chan WCW. Determining the size and shape dependence of gold nanoparticle uptake into mammalian cells. Nano Lett. (2006) 6:662–8. 10.1021/nl052396o 16608261

[35] Yue J Feliciano TJ Li W Lee A Odom TW. Gold nanoparticle size and shape effects on cellular uptake and intracellular distribution of siRNA nanoconstructs. Bioconjug Chem. (2017) 28:1791–800. 10.1021/acs.bioconjchem.7b00252 28574255 PMC5737752

[36] Da Silva-Candal Brown T Krishnan V Lopez-Loureiro I Avila-Gomez P Pusuluri A. Shape effect in active targeting of nanoparticles to inflamed cerebral endothelium under static and flow conditions. J Control Release. (2019) 309:94–105. 10.1016/j.jconrel.2019.07.026 31330214

[37] Salatin S Maleki Dizaj S, A. Yari Khosroushahi. Effect of the surface modification, size, and shape on cellular uptake of nanoparticles. Cell Biol Int. (2015) 39:881–90. 10.1002/cbin.10459 25790433

[38] Yang K Ma YQ. Computer simulation of the translocation of nanoparticles with different shapes across a lipid bilayer. Nat Nanotechnol. (2010) 5:579–83. 10.1038/nnano.2010.141 20657599

[39] Xiong K Zhao J Yang D Cheng Q Wang J Ji H. Cooperative wrapping of nanoparticles of various sizes and shapes by lipid membranes. Soft Matter. (2017) 13:4644–52. 10.1039/C7SM00345E 28650048

[40] Dasgupta S Auth T Gompper G. Shape and orientation matter for the cellular uptake of nonspherical particles. Nano Lett. (2014) 14:687–93. 10.1021/nl403949h 24383757

[41] Vacha R Martinez-Veracoechea FJ Frenkel D. Receptor-mediated endocytosis of nanoparticles of various shapes. Nano Lett. (2011) 11:5391–5. 10.1021/nl2030213 22047641

[42] Verma A Stellacci F. Effect of surface properties on nanoparticle-cell interactions. Small. (2010) 6:12–21. 10.1002/smll.200901158 19844908

[43] Gratton SEA Ropp PA Pohlhaus PD Luft JC Madden VJ Napier ME. The effect of particle design on cellular internalization pathways. Proc Nat Acad Sci U S A. (2008) 105:11613. 10.1073/pnas.0801763105 18697944 PMC2575324

[44] Yahya I Eltayeb M. Modeling of nano-carriers for vascular-targeted delivery for blood clots treatment. BioRxiv [Preprint]. (2020). 10.1101/2020.07.02.184242

[45] Nangia S Sureshkumar R. Effects of nanoparticle charge and shape anisotropy on translocation through cell membranes. Langmuir. (2012) 28:17666–71. 10.1021/la303449d 23088323

[46] Li Y Yuan B Yang K Zhang X Yan B Cao D. Counterintuitive cooperative endocytosis of like-charged nanoparticles in cellular internalization: computer simulation and experiment. Nanotechnology. (2017) 28:085102. 10.1088/1361-6528/aa56e0 28054516

[47] Shen Z Ye H Li Y. Understanding receptor-mediated endocytosis of elastic nanoparticles through coarse grained molecular dynamic simulation. Phys Chem Chem Phys. (2018) 20:16372–16385. 10.1039/C7CP08644J 29445792

[48] Shen Z Ye H Yi X Li Y. Membrane wrapping efficiency of elastic nanoparticles during endocytosis: size and shape matter. ACS Nano. (2019) 13:215–28. 10.1021/acsnano.8b05340 30557506

[49] Ding HM Ma YQ. Interactions between Janus particles and membranes. Nanoscale. (2012) 4:1116–22. 10.1039/C1NR11425E 22116542

[50] Li Y Chen X Gu N. Computational investigation of interaction between nanoparticles and membranes: hydrophobic/hydrophilic effect. J Phys Chem B. (2008) 112:16647–53. 10.1021/jp8051906 19032046

[51] Gupta R Rai B. *In-silico* design of nanoparticles for transdermal drug delivery application. Nanoscale. (2018) 10:4940–51. 10.1039/C7NR07898F 29485168

[52] Burello E. Computational design of safer nanomaterials. Environ Sci Nano. (2015) 2:454–62. 10.1039/C5EN00066A

[53] Moseley P Curtin WA. Computational design of strain in core-shell nanoparticles for optimizing catalytic activity. Nano Lett. (2015) 15:4089–95. 10.1021/acs.nanolett.5b01154 25965405

[54] Price LSL Stern ST Deal AM Kabanov AV Zamboni WC. A reanalysis of nanoparticle tumor delivery using classical pharmacokinetic metrics. Sci Adv. (2020) 6:eaay9249. 10.1126/sciadv.aay9249 32832614 PMC7439617

[55] Beg S Jain S Kushwah V Bhatti GK Sandhu PS Katare OP. Novel surface-engineered solid lipid nanoparticles of rosuvastatin calcium for low-density lipoprotein-receptor targeting: a Quality by Design-driven perspective. Nanomedicine. (2017) 12:333–56. 10.2217/nnm-2016-0336 28093941

[56] Wilhelm S Tavares AJ Dai Q Ohta S Audet J Dvorak HF. Analysis of nanoparticle delivery to tumours. Nat Rev Mater. (2016) 1:16014. 10.1038/natrevmats.2016.14

[57] Abraham MJ Murtola T Schulz R Páll S Smith JC Hess B. GROMACS: high performance molecular simulations through multi-level parallelism from laptops to supercomputers. SoftwareX. (2015) 1–2:19–25. 10.1016/j.softx.2015.06.001

[58] Hess B Kutzner C van der Spoel D Lindahl E. GROMACS 4: algorithms for highly efficient, load-balanced, and scalable molecular simulation. J Chem Theory Comput. (2008) 4:435–47. 10.1021/ct700301q 26620784

[59] Waclaw B Bozic I Pittman ME Hruban RH Vogelstein B Nowak MA. A spatial model predicts that dispersal and cell turnover limit intratumour heterogeneity. Nature. (2015) 525:261–4. 10.1038/nature14971 26308893 PMC4782800

[60] Izaguirre JA Chaturvedi R Huang C Cickovski T Coffland J Thomas G. CompuCell, a multi-model framework for simulation of morphogenesis. Bioinformatics. (2004) 20:1129–37. 10.1093/bioinformatics/bth050 14764549

[61] Swat MH Thomas GL Belmonte JM Shirinifard A Hmeljak D Glazier JA. Multi-scale modeling of tissues using CompuCell3D. Methods Cell Biol. (2012) 110:325–66. 10.1016/B978-0-12-388403-9.00013-8 22482955 PMC3612985

[62] Mirams GR Arthurs CJ Bernabeu MO Bordas R Cooper J Corrias A. Chaste: an open source C++ library for computational physiology and biology. PLoS Comput Biol. (2013) 9:e1002970. 10.1371/journal.pcbi.1002970 23516352 PMC3597547

[63] Pitt-Francis J Pathmanathan P Bernabeu MO Bordas R Cooper J Fletcher AG. Chaste: a test-driven approach to software development for biological modelling. Comput Phys Commun. (2009) 180:2452–71. 10.1016/j.cpc.2009.07.01918565813

[64] Iwasaki WM Innan H. Simulation framework for generating intratumor heterogeneity patterns in a cancer cell population. PLoS One. (2017) 12:e0184229. 10.1371/journal.pone.0184229 28877206 PMC5587296

[65] Hoehme S Drasdo D. A cell-based simulation software for multi-cellular systems. Bioinformatics. (2010) 26:2641–2. 10.1093/bioinformatics/btq437 20709692 PMC2951083

[66] Ghaffarizadeh A Heiland R Friedman SH Mumenthaler SM Macklin P. PhysiCell: an open source physics-based cell simulator for 3-D multicellular systems. PLoS Comput Biol. (2018) 14:e1005991. 10.1371/journal.pcbi.1005991 29474446 PMC5841829

[67] Kang S Kahan S McDermott J Flann N Shmulevich I. Biocellion: accelerating computer simulation of multicellular biological system models. Bioinformatics. (2014) 30:3101–8. 10.1093/bioinformatics/btu498 25064572 PMC4609016

[68] Rejniak KA. An immersed boundary framework for modelling the growth of individual cells: an application to the early tumour development. J Theor Biol. (2007) 247:186–204. 10.1016/j.jtbi.2007.02.019 17416390

[69] Tanaka S Sichau D Iber D. LBIBCell: a cell-based simulation environment for morphogenetic problems. Bioinformatics. (2015) 31:2340–7. 10.1093/bioinformatics/btv147 25770313

[70] Rejniak KA Wang SE Bryce NS Chang H Parvin B Jourquin J. Linking changes in epithelial morphogenesis to cancer mutations using computational modeling. PLoS Comput Biol. (2010) 6:e1000900. 10.1371/journal.pcbi.1000900 20865159 PMC2928778

[71] Andrews SS. Smoldyn: particle-based simulation with rule-based modeling, improved molecular interaction and a library interface. Bioinformatics. (2017) 33:710–7. 10.1093/bioinformatics/btw700 28365760

[72] Andrews SS. Spatial and stochastic cellular modeling with the Smoldyn simulator. Methods Mol Biol. (2012) 804:519–42. 10.1007/978-1-61779-361-5_26 22144170

[73] Chen W De Schutter E. Parallel STEPS: large scale stochastic spatial reaction-diffusion simulation with high performance computers. Front Neuroinform. (2017) 11:13. 10.3389/fninf.2017.00013 28239346 PMC5301017

[74] Hepburn I Chen W Wils S De Schutter E. STEPS: efficient simulation of stochastic reaction-diffusion models in realistic morphologies. BMC Syst Biol. (2012) 6:36. 10.1186/1752-0509-6-36 22574658 PMC3472240

[75] Drawert B Engblom S Hellander A. URDME: a modular framework for stochastic simulation of reaction-transport processes in complex geometries. BMC Syst Biol. (2012) 6:76. 10.1186/1752-0509-6-76 22727185 PMC3439286

[76] Starruss J. de Back W Brusch L Deutsch A. Morpheus: a user-friendly modeling environment for multiscale and multicellular systems biology. Bioinformatics. (2014) 30:1331–2. 10.1093/bioinformatics/btt772 24443380 PMC3998129

[77] Loew LM Schaff JC. The Virtual Cell: a software environment for computational cell biology. Trends Biotechnol. (2001) 19:401–6. 10.1016/S0167-7799(01)01740-1 11587765

[78] Moraru I Schaff JC Slepchenko BM Blinov ML Morgan F Lakshminarayana A. Virtual cell modelling and simulation software environment. IET Syst Biol. (2008) 2:352–62. 10.1049/iet-syb:20080102 19045830 PMC2711391

[79] Palchetti S Colapicchioni V Digiacomo L Caracciolo G Pozzi D Capriotti AL. The protein corona of circulating PEGylated liposomes. Biochim Biophys Acta. (2016) 1858:189–96. 10.1016/j.bbamem.2015.11.012 26607013

[80] Xiao W Gao H. The impact of protein corona on the behavior and targeting capability of nanoparticle-based delivery system. Int J Pharm. (2018) 552:328–39. 10.1016/j.ijpharm.2018.10.011 30308270

[81] Lee H. Molecular modeling of protein corona formation and its interactions with nanoparticles and cell membranes for nanomedicine applications. Pharmaceutics. (2021) 13:637. 10.3390/pharmaceutics13050637 33947090 PMC8145147

[82] Ramezani F Rafii-Tabar H. An in-depth view of human serum albumin corona on gold nanoparticles. Mol Biosyst. (2015) 11:454–62. 10.1039/C4MB00591K 25409650

[83] Hu G Jiao B Shi X Valle RP Fan Q Zuo YY. Physicochemical properties of nanoparticles regulate translocation across pulmonary surfactant monolayer and formation of lipoprotein corona. ACS Nano. (2013) 7:10525–33. 10.1021/nn4054683 24266809 PMC5362675

[84] Lopez H Lobaskin V. Coarse-grained model of adsorption of blood plasma proteins onto nanoparticles. J Chem Phys. (2015) 143:243138. 10.1063/1.4936908 26723623

[85] Pal K Al-Suraih F Gonzalez-Rodriguez R Dutta SK Wang E Kwak HS. Multifaceted peptide assisted one-pot synthesis of gold nanoparticles for plectin-1 targeted gemcitabine delivery in pancreatic cancer. Nanoscale. (2017) 9:15622–34. 10.1039/C7NR03172F 28991294 PMC5859336

[86] Lee TR Choi M Kopacz AM Yun SH Liu WK Decuzzi P. On the near-wall accumulation of injectable particles in the microcirculation: smaller is not better. Sci Rep. (2013) 3:2079. 10.1038/srep02079 23801070 PMC3693098

[87] Muller K Fedosov DA Gompper G. Margination of micro- and nano-particles in blood flow and its effect on drug delivery. Sci Rep. (2014) 4:4871. 10.1038/srep04871 24786000 PMC4007071

[88] Vizirianakis IS Mystridis GA Avgoustakis K Fatouros DG Spanakis M. Enabling personalized cancer medicine decisions: the challenging pharmacological approach of PBPK models for nanomedicine and pharmacogenomics (Review). Oncol Rep. (2016) 35:1891–904. 10.3892/or.2016.4575 26781205

[89] Bachler G von Goetz N Hungerbuhler K. A physiologically based pharmacokinetic model for ionic silver and silver nanoparticles. Int J Nanomedicine. (2013) 8:3365–82. 10.2147/IJN.S46624 24039420 PMC3771750

[90] Betker JL Anchordoquy TJ. The use of lactose as an alternative coating for nanoparticles. J Pharm Sci. (2020) 109:1573–1580. 10.1016/j.xphs.2020.01.019 32004536 PMC7096283

[91] Chu D Gao J Wang Z. Neutrophil-mediated delivery of therapeutic nanoparticles across blood vessel barrier for treatment of inflammation and infection. ACS Nano. (2015) 9:11800–11. 10.1021/acsnano.5b05583 26516654 PMC4699556

[92] Dong X Chu D Wang Z. Leukocyte-mediated delivery of nanotherapeutics in inflammatory and tumor sites. Theranostics. (2017) 7:751–63. 10.7150/thno.18069 28255364 PMC5327647

[93] Chaudhari KR Ukawala M Manjappa AS Kumar A Mundada PK Mishra AK. Opsonization, biodistribution, cellular uptake and apoptosis study of PEGylated PBCA nanoparticle as potential drug delivery carrier. Pharm Res. (2012) 29:53–68. 10.1007/s11095-011-0510-x 21744174

[94] Hayat SMG Jaafari MR Hatamipour M Penson PE Sahebkar A. Liposome circulation time is prolonged by CD47 coating. Protein Pept Lett. (2020) 27:1029–37. 10.2174/0929866527666200413100120 32282292

[95] Zhang Y Liu AT Cornejo YR Van Haute D Berlin JM. A Systematic comparison of *in vitro* cell uptake and *in vivo* biodistribution for three classes of gold nanoparticles with saturated PEG coatings. PLoS One. (2020) 15:e0234916. 10.1371/journal.pone.0234916 32614882 PMC7332061

[96] Suk JS Xu Q Kim N Hanes J Ensign LM. PEGylation as a strategy for improving nanoparticle-based drug and gene delivery. Adv Drug Deliv Rev. (2016) 99:28–51. 10.1016/j.addr.2015.09.012 26456916 PMC4798869

[97] Moradi Kashkooli F Soltani M Souri M Meaney C Kohandel M. Nexus between *in silico* and *in vivo* models to enhance clinical translation of nanomedicine. Nano Today. (2021) 36:101057. 10.1016/j.nantod.2020.101057

[98] Shah PN Lin TY Aanei IL Klass SH Smith BR Shaqfeh ESG. Extravasation of Brownian spheroidal nanoparticles through vascular pores. Biophys J. (2018) 115:1103–15. 10.1016/j.bpj.2018.07.038 30201266 PMC6139985

[99] Subhan MA Yalamarty SS Filipczak N Parveen F Torchilin VP. Recent advances in tumor targeting *via* EPR effect for cancer treatment. J Pers Med. (2021) 11:571. 10.3390/jpm11060571 34207137 PMC8234032

[100] LoCastro E Paudyal R Mazaheri Y Hatzoglou V Oh JH Lu Y. Computational modeling of interstitial fluid pressure and velocity in head and neck cancer based on dynamic contrast-enhanced magnetic resonance imaging: feasibility analysis. Tomography. (2020) 6:129–38. 10.18383/j.tom.2020.00005 32548289 PMC7289251

[101] Qian J Rankin EB. Hypoxia-induced phenotypes that mediate tumor heterogeneity. Adv Exp Med Biol. (2019) 1136:43–55. 10.1007/978-3-030-12734-3_3 31201715 PMC7039393

[102] Powathil G Kohandel M Milosevic M Sivaloganathan S. Modeling the spatial distribution of chronic tumor hypoxia: implications for experimental and clinical studies. Comput Math Methods Med. (2012) 2012:410602. 10.1155/2012/410602 22400049 PMC3287099

[103] Welter M Rieger H. Interstitial fluid flow and drug delivery in vascularized tumors: a computational model. PLoS One. (2013) 8:e70395. 10.1371/journal.pone.0070395 23940570 PMC3734291

[104] Wang HF Ran R Liu Y Hui Y Zeng B Chen D. Tumor-vasculature-on-a-chip for investigating nanoparticle extravasation and tumor accumulation. ACS Nano. (2018) 12:11600–9. 10.1021/acsnano.8b06846 30380832

[105] Vu MN Rajasekhar P Poole DP Khor SY Truong NP Nowell CJ. Rapid assessment of nanoparticle extravasation in a microfluidic tumor model. ACS Appl Nano Mater. (2019) 2:1844–56. 10.1021/acsanm.8b02056

[106] Curtis LT Wu M Lowengrub J Decuzzi P Frieboes HB. Computational modeling of tumor response to drug release from vasculature-bound nanoparticles. PLoS One. (2015) 10:e0144888. 10.1371/journal.pone.0144888 26660469 PMC4682796

[107] Meacham CE Morrison SJ. Tumour heterogeneity and cancer cell plasticity. Nature. (2013) 501:328–37. 10.1038/nature12624 24048065 PMC4521623

[108] Van Liedekerke P Palm MM Jagiella N Drasdo D. Simulating tissue mechanics with agent-based models: concepts, perspectives and some novel results. Comput Part Mech. (2015) 2:401–44. 10.1007/s40571-015-0082-3

[109] Van Liedekerke P Buttenschön A Drasdo D. Chapter 14 - Off-lattice agent-based models for cell and tumor growth: numerical methods, implementation, and applications. In: Cerrolaza M. Shefelbine SJ Garzón-Alvarado D editors. Numerical Methods and Advanced Simulation in Biomechanics and Biological Processes. Academic Press (2018). p. 245–67.

[110] Tsompanas MA Bull L Adamatzky A Balaz I. *In silico* optimization of cancer therapies with multiple types of nanoparticles applied at different times. Comput Methods Programs Biomed. (2021) 200:105886. 10.1016/j.cmpb.2020.105886 33288217

[111] Gong C Zhang X Shi M Li F Wang S Wang Y. Tumor exosomes reprogrammed by low pH are efficient targeting vehicles for smart drug delivery and personalized therapy against their homologous tumor. Adv Sci (Weinh). (2021) 8:2002787. 10.1002/advs.202002787 34026432 PMC8132050

[112] Henke E Nandigama R Ergun S. Extracellular matrix in the tumor microenvironment and its impact on cancer therapy. Front Mol Biosci. (2019) 6:160. 10.3389/fmolb.2019.00160 32118030 PMC7025524

[113] Hou L Chen D Hao L Tian C Yan Y Zhu L. Transformable nanoparticles triggered by cancer-associated fibroblasts for improving drug permeability and efficacy in desmoplastic tumors. Nanoscale. (2019) 11:20030–44. 10.1039/C9NR06438A 31612175

[114] Zhang Y Elechalawar CK Hossen MN Francek ER Dey A Wilhelm S. Gold nanoparticles inhibit activation of cancer-associated fibroblasts by disrupting communication from tumor and microenvironmental cells. Bioact Mater. (2021) 6:326–32. 10.1016/j.bioactmat.2020.08.009 32954051 PMC7479257

[115] Behzadi S Serpooshan V Tao W Hamaly MA Alkawareek MY Dreaden EC. Cellular uptake of nanoparticles: journey inside the cell. Chem Soc Rev. (2017) 46:4218–44. 10.1039/C6CS00636A 28585944 PMC5593313

[116] Yameen B Choi WI Vilos C Swami A Shi J Farokhzad OC. Insight into nanoparticle cellular uptake and intracellular targeting. J Control Release. (2014) 190:485–99. 10.1016/j.jconrel.2014.06.038 24984011 PMC4153400

[117] Qu ZG He XC Lin M Sha BY Shi XH Lu TJ. Advances in the understanding of nanomaterial-biomembrane interactions and their mathematical and numerical modeling. Nanomedicine. (2013) 8:995–1011. 10.2217/nnm.13.81 23730698

[118] Roiter Y Ornatska M Rammohan AR Balakrishnan J Heine DR Minko S, Interaction Interaction of nanoparticles with lipid membrane. Nano Lett. (2008) 8:941–4. 10.1021/nl080080l 18254602

[119] Roiter Y Ornatska M Rammohan AR Balakrishnan J Heine DR Minko S. Interaction of lipid membrane with nanostructured surfaces. Langmuir. (2009) 25:6287–99. 10.1021/la900119a 19466783

[120] Zhao Y Sun X Zhang G Trewyn BG Slowing II Lin VSY. Interaction of mesoporous silica nanoparticles with human red blood cell membranes: size and surface effects. ACS Nano. (2011) 5:1366–75. 10.1021/nn103077k 21294526

[121] Kostarelos K Lacerda L Pastorin G Wu W Wieckowski S Luangsivilay J. Cellular uptake of functionalized carbon nanotubes is independent of functional group and cell type. Nat Nanotechnol. (2007) 2:108–13. 10.1038/nnano.2006.209 18654229

[122] Leroueil PR Berry SA Duthie K Han G Rotello VM McNerny DQ. Wide varieties of cationic nanoparticles induce defects in supported lipid bilayers. Nano Lett. (2008) 8:420–4. 10.1021/nl0722929 18217783

[123] Cho EC Xie J Wurm PA Xia Y. Understanding the role of surface charges in cellular adsorption versus internalization by selectively removing gold nanoparticles on the cell surface with a I2/KI etchant. Nano Lett. (2009) 9:1080–4. 10.1021/nl803487r 19199477

[124] Canton I Battaglia G. Endocytosis at the nanoscale. Chem Soc Rev. (2012) 41:2718–39. 10.1039/c2cs15309b 22389111

[125] Huang C Zhang Y Yuan H Gao H Zhang S. Role of nanoparticle geometry in endocytosis: laying down to stand up. Nano Lett. (2013) 13:4546–50. 10.1021/nl402628n 23972158

[126] Ding HM Ma YQ. Role of physicochemical properties of coating ligands in receptor-mediated endocytosis of nanoparticles. Biomaterials. (2012) 33:5798–802. 10.1016/j.biomaterials.2012.04.055 22607914

[127] Ding H Tian W Ma Y. Designing nanoparticle translocation through membranes by computer simulations. ACS Nano. (2012) 6:1230–38. 10.1021/nn2038862 22208867

